# Di-(2-ethylhexyl) phthalate-induced tumor growth is regulated by primary cilium formation via the axis of H_2_O_2_ production-thymosin beta-4 gene expression

**DOI:** 10.7150/ijms.53595

**Published:** 2021-01-14

**Authors:** Jae-Wook Lee, Pham Xuan Thuy, Hae-Kyoung Han, Eun-Yi Moon

**Affiliations:** Department of Bioscience and Biotechnology, Sejong University, Seoul 05006, Republic of Korea.

**Keywords:** DEHP, Thymosin beta-4(TB4), ROS, primary cilium (PC), tumor growth

## Abstract

Di-(2-ethylhexyl) phthalate (DEHP) that is one of the most commonly used phthalates in manufacturing plastic wares regulates tumorigenesis. Thymosin beta-4 (TB4), an actin-sequestering protein, has been reported as a novel regulator to form primary cilia that are antenna-like organelles playing a role in various physiological homeostasis and pathological development including tumorigenesis. Here, we investigated whether DEHP affects tumor growth via primary cilium (PC) formation via the axis of TB4 gene expression and the production of reactive oxygen species (ROS). Tumor growth was increased by DEHP treatment that enhanced TB4 expression, PC formation and ROS production. The number of cells with primary cilia was enhanced time-dependently higher in HeLa cells incubated in the culture medium with 0.1% fetal bovine serum (FBS). The number of cells with primary cilia was decreased by the inhibition of TB4 expression. The incubation of cells with 0.1% FBS enhanced ROS production and the transcriptional activity of TB4 that was reduced by ciliobrevin A (CilioA), the inhibitor of ciliogenesis. ROS production was decreased by catalase treatment but not by mito-TEMPO, which affected to PC formation with the same trend. H_2_O_2_ production was reduced by siRNA-based inhibition of TB4 expression. H_2_O_2_ also increased the number of ciliated cells, which was reduced by siRNA-TB4 or the co-incubation with CilioA. Tumor cell viability was maintained by ciliogenesis, which was correlated with the changes of intracellular ATP amount rather than a simple mitochondrial enzyme activity. TB4 overexpression enhanced PC formation and DEHP-induced tumor growth. Taken together, data demonstrate that DEHP-induced tumor growth might be controlled by PC formation via TB4-H_2_O_2_ axis. Therefore, it suggests that TB4 could be a novel bio-marker to expect the risk of DEHP on tumor growth.

## Introduction

Primary cilia are antenna-like microtubule-based organelles protruded from plasma membrane surface in most vertebrate cells. The oldest primary cilia were first observed in protozoa by Anthony van Leeuwenhoek in 1675 with description by 'incredibly thin little feet, or little legs, which were moved very nimbly' 1. Since primary cilia transduced diverse intracellular signaling involved in embryonic development and tissue homeostasis, primary cilium (PC) abnormality is major causes of development disorders and human diseases such as cancers 2-4. PC biogenesis is induced by the incubation with serum-starved medium (0.5% FBS) 5-7 in many types of cells 5, 8. Under serum-starved conditions, the production of reactive oxygen species (ROS) could be increased by the incomplete mitochondrial oxidative phosphorylation 9, which led to apoptotic cell death 10.

ROS include free radicals such as superoxide (O_2_**˙**ˉ), hydroxyl radical (**˙**OH), and nitric oxide (NO**˙**), and non-radical molecules including hydrogen peroxide (H_2_O_2_) and highly reactive carbonyl compounds 11. ROS are not only produced by normal mitochondrial oxidative metabolism, but also enhanced by exogenous sources including xenobiotics, cytokines, and bacterial infection and by various disease states including cancer, diabetes, aging, atherosclerosis, and neurodegeneration 12, 13. However, little has been known about molecular linkers between PC biogenesis and tumor cell changes by ROS.

Thymosin beta-4 (TB4), actin-sequestering protein, is highly conserved, naturally occurring 43-amino acid peptide, which is ubiquitously expressed in almost all type of cells with exception of erythrocytes 14-17. TB4 plays various roles in angiogenesis 18-21, tumor cell growth 22-25, survival 26, 27, migration 14, 28, 29, metastasis 30-33 and drug resistance 34, 35 in many types of cancer cells. TB4 has been also reported as a novel regulator to form primary cilia 36. In addition, TB4 plays various different roles in controlling ROS-mediated cellular functions, which are dependent on specific conditions including extracellular stimulants and types of tissues or cells. TB4 pre-treatment reduces oxidative stress in cardiomyocytes and human cornea epithelial cells by upregulating antioxidant enzymes such as manganese superoxide dismutase (SOD), cooper/zinc SOD and catalase 37, 38. Myotubes exposed to extrinsic H_2_O_2_ decreased actin polymerization by downregulating TB4 39. On the other hands, TB4 increase ROS level to stabilize HIF-1α in HeLa cells 35. However, little has been known about whether TB4 could be a possible molecule to link PC formation and ROS production.

Di-(2-ethylhexyl) phthalate (DEHP) is the most common phthalate used in manufacturing of various polyvinyl chloride (PVC)-containing products (ATSDR, 2009; Rusyn et al., 2006). DEHP is one of endocrine disruptors and acts as xenoestrogen which shows reproductive, developmental, and carcinogenic toxicity on both animal and human health 40-44. DEHP also promotes cancer cell metastasis 45, 1,2-dimethylhydrazine (DMH)-induced colon tumorigenesis 46 and hepatic tumorigenesis 47, 48. Oxidative stress is one of the important mechanisms on the toxicity of DEHP 49, which lead to hepatotoxicity by lipid peroxidation 50. In the meanwhile, endocrine disruptor, cadmium, induced cytoskeleton disruption leading to apoptotic cell death 51. However, little is known about whether endocrine disruptor, DEHP, might affect tumor growth by the regulation of cytoskeletal microtubule-based PC formation via TB4-ROS axis.

In this study, we investigated whether TB4 could be a possible molecule to link PC formation and ROS production. We also investigated whether DEHP might affect tumor growth by the regulation of PC formation via TB4-ROS axis. Our data demonstrate that PC formation was controlled by TB4 through ROS production. It suggests that TB4 could be a possible molecule to link PC formation and ROS production leading to tumor cell changes. Our data also showed that DEHP could augment tumor growth by TB4 overexpression and the increase in PC formation via TB4-ROS axis. So, it suggests that the individual with a higher level of TB4 could be very vulnerable to tumor growth by the exposure repeatedly to DEHP.

## Materials and Methods

### Mice and reagents

Six weeks old male C57BL/6(H-2^b^) mice were obtained from Daehan BioLink (Chungju, Republic of Korea). Five mice were housed in the transparent acrylic cage and maintained in the pathogen-free authorized facility in Sejong University where the temperature was at 20-22 °C, the humidity at 50-60%, and a dark/light cycle at 12 h. Mouse-used all experiments were carried out in strict accordance with the guidelines by the recommendations in the Guide for the Care and Use of Laboratory Animals of 'Animal and Plant Quarantine Agency', Republic of Korea. The protocol was approved by the Institutional Animal Care and Use Committee, Sejong University (Permit Number: SJ20160702). All efforts were made to minimize suffering animals.

DEHP, N-acetyl-L-cysteine (NAC), hydrogen peroxide (H_2_O_2_), MTT [3(4,5-dimethyl-thiazol-2-yl)-2,5-diphenyl tetrazolium bromide] and 4′,6-diamidno-2-phenylinole(DAPI) were purchased from the Sigma Chemical Co. (St. Louis, MO, USA). 2',7'-dichlorofluorescin diacetate (DCF-DA) was purchased from Molecular Probe (Eugene, Oregon, USA). Mouse antibodies which are reactive with acetylated tubulin (T7451), and β-tubulin (T4026) were from Sigma-Aldrich Co. (St. Louis, MO, USA). Rabbit antibodies which are reactive with GFP (sc-138) were from Santa Cruz Biotechnology, Inc (Santa Cruz, CA, USA). Chicken anti-mouse IgG-Alexa 488 (A-21200) were obtained from Invitrogen (Calsbad, CA, USA). Except where indicated, all other materials are obtained from the Sigma Chemical Co. (St. Louis, MO, USA).

### Plasmids and siRNAs

Plasmids, pCDNA3.1 and pCMV-2B were kindly provided by Prof. Young-Joo Jang, College of Dentistry, Dankook University (Cheon-An, Rep. of Korea). pEGFP-C2 plasmid was kindly provided by Prof. Mi-Ock Lee, College of Pharmacy, Seoul National University (Seoul, Rep. of Korea). pCMV-TB4 and pEGFP-C2-TB4 plasmids were generated by customer order for subcloning to Cosmo Genetech Co., Ltd. (Seoul, Rep. of Korea).

Small interference(si) RNAs are customer-ordered to Bioneer (Daejeon, Rep. of Korea). Sequences of siRNAs for TB4 are as follows; sense: CCG AUA UGG CUG AGA A; anti-sense: UCG AUC UCA GCC AUA UCG G. AccuTarget™ negative control siRNA (SN-1001) was also purchased from Bioneer (Daejeon, Rep. of Korea).

### Cell culture

HeLa human cervical cancer cells (ATCC # CCL-2), human embryonic kidney (HEK) 293T cells and B16F10 mouse melanoma cells were obtained from Korea research institute of bioscience and biotechnology (KRIBB) cell bank (Daejeon, Rep.of Korea). Cells were cultured as monolayers in Dullecco's modified Eagle's medium (DMEM) with supplement of 10% fetal bovine serum (FBS) (GIBCO, Grand Island, NY, USA), 2 mM L-glutamine, 100 units/ml penicillin and streptomycin (GIBCO, Grand Island, NY, USA). Cells were incubated at 37 °C in a humidified atmosphere of 5% CO_2_ maintenance. For the induction of PC formation, cells were incubated in serum-starved media with 0.1% FBS for 36 h.

### *In vivo* exposures of DEHP to mice

For the preparation of PECs or BMDMs from DEHP-treated mice *in vivo*, DEHP was suspended in normal saline to yield 0.4 mg/ml stock solution. Prior to the usage for injection, the suspensions underwent sonication to assure no significant agglomeration in the aqueous solution. Immediately after sonication step, mouse was intraperitoneally injected with DEHP suspension for 21 or 28 days. Each animal was weighed daily and the amount of saline needed to inject the stock solution was adjusted accordingly based on body weight values (100 μl per 10 g body weight). A final dose of DEHP became 4.0 mg/kg.

### *In vivo* tumor growth

The effect of DEHP on *in vivo* tumor growth was observed by using B16F10 mouse melanoma tumor xenograft model. DEHP suspension was daily prepared by the method described in above and intraperitoneally injected into mice (*n* = 5 per group) at the dose of 4.0 mg/kg DEHP for 21 days before the implantation of B16F10 tumor cells. Then, mice were subcutaneously implanted with 2×10^5^ cells B16F10 mouse melanoma cells. Mice were intraperitoneally injected with 4.0 mg/kg DEHP for an additional 7 days after B16F10 tumor cell implantation. Tumor nodule formation was checked by the tip of index finger from the next day of tumor cell implantation. Tumor growth was monitored for 14 days by the measurement of short axis and long axis of tumor mass with Vernier calipers (Mititoyo, Mizuyo, Japan). Tumor volume was calculated by the multiplication of the [(long axis) / 2] by the (short axis)^2^.

### Reverse transcription polymerase chain reaction (RT-PCR)

Total RNA was extracted by using TRizol reagent (Invitrogen, Calsbad, CA, USA). Complementary DNA (cDNA) was synthesized from 1 μg of isolated total RNA, oligo-dT_18_, and superscript reverse transcriptase (Bioneer, Daejeon, Rep. of Korea) in a final volume of 20 μl. For standard PCR, 1 μl of template cDNA was amplified with Taq DNA polymerase. PCR amplification was performed with 25 ~ 35 thermocycles for 30 sec at 95 °C, 30 sec at 55 °C, and 60 sec at 72 °C using human (h) or mouse (m) oligonucleotide primers specific for hTB4 (sense: ACA AAC CCG ATA TGG CTG AG; anti-sense: CCT CCA AGG AAG AGA CTG AA), mTB4 (sense: ATA TGG CTG AGA TCG AGA AA; anti-sense: GCT TGC TTC TCT TGT TCA AT), hGAPDH (sense: GAA GGT GAA GGT CGG AGT C; anti-sense: GAA GAT GGT GAT GGG ATT TC) and mGAPDH (sense: TCC ACC ACC CTG TTG CTG TA; anti-sense: ACC ACA GTC CAT GCC ATC AC). Amplified PCR products were separated by 1.0 ~ 1.5% agarose gel electrophoresis and detected on Ugenius 3^®^ gel documentation system (Syngene, Cambridge, United Kingdom).

### Detection of primary cilia

For the detection of primary cilia *in vitro*, cells were maintained in serum-starved culture medium for 24-72 h 52-55. Briefly, HeLa cells were grown on coverslip and then incubated with serum-starved DMEM with 0.1% FBS for 36 h. Cells were fixed with 4% paraformaldehyde for 10 min, washed three times with cold PBS, and permeabilized with PBST (0.1% (v/v) Triton X-100 in PBS) for 10 min. Then, cells were washed three times, and incubated with monoclonal anti-acetylated tubulin antibodies diluted (1:1000) in PBST for 1 h at room temperature. After washing three times with PBS, cells were incubated with chicken anti-mouse IgG-Alexa 488 diluted (1:1000) in PBST for 1h at room temperature. Nucleus was visualized by staining cells with DAPI. After washing with PBS, cells were mounted on glass slide. Primary cilia were observed and photographed at 1000× magnification under a fluorescence microscope (Nikon, Tokyo, Japan).

### Cytotoxicity assay

Cell survival was quantified by using colorimetric assay with MTT to measure intracellular succinate dehydrogenase content or by using luminescence assay with CellTiter-Glo substrate to measure intracellular ATP content 56. For MTT assay, confluent cells were cultured with various concentrations of each reagent for 24 h. Cells were then incubated with 50 μg/ml of MTT at 37 °C for 2 h. Formazan formed by MTT were dissolved in dimethylsulfoxide (DMSO). Optical density (OD) was read at 540 nm. For CellTiter-Glo assay, cell cultures were treated with CellTiter-Glo substrate (Promega, Madison, WI). Luminescence was detected by using Lumet 3, LB9508 tube luminometer (Berthold Technologies GmbH & Co. KG, Bad Wildbad, Germany).

### ROS measurement

To measure reactive oxygen species (ROS), intracellular ROS level was measured by incubating cells with or without 10 μM DCF-DA at 37 °C for 30 min. Fluorescence intensity of 10,000 cells was analysed by FACSCalibur™ (Becton Dickinson, San Joes, CA, USA).

### H_2_O_2_ measurement

The rate of H_2_O_2_ release was measured by the changes in fluorescence of scopoletin as reported previously 57. Fluorescent scopoletin is changed into non-fluorescent materials by H_2_O_2_ production during the incubation. Briefly, cells were incubated with LPS in the presence or absence of various concentrations of DEHP for 6 or 18 h. The cultures were washed 3 times with PBS. The assay mixture (prewarmed to 37 °C) was prepared immediately before use from stock solutions and consisted of 30 μM scopoletin, 1 mM NaN_3_, in KRPG supplemented with 5.5 mM glucose. Krebs-Ringer phosphate buffer (KRP) was 129 mM NaCl, 4.86 mM KCl, 0.54 mM CaCl_2_, 1.22 mM MgSO_4_, 15.8 mM sodium phosphate, pH 7.35, 300-315 mosM. Scopoletin was prepared as a 1 mM solution in KRP by dissolution for 24 h at 37 °C, sterile filtered and stored at 4 °C in the dark. Immediately after the addition of the assay mixture into the wells (100 μl/well), the fluorescence was read in microplate fluorometer (SpecraFluor plus, TECAN, Alexandria, Austria) with the excitation at 360 nm and the emission at 460 nm. Then, the plate was transferred to the 37 °C incubation chamber and maintained for 60 min. The fluorescence (F) in each well was again recorded and the data were expressed as below.


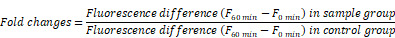


### Transfection of nucleic acids

Each plasmid DNA, siRNAs for TB4 and AccuTarget™ negative contol siRNA were transfected into cells as follows 36. Briefly, each nucleic acid and lipofectamine 2000 (Invitrogen, Calsbad, CA, USA) was diluted in serum-free medium and incubated for 5 min, respectively. The diluted nucleic acid and lipofectamine 2000 reagent was mixed by inverting and incubated for 20 min to form complexes. In the meanwhile, cells were stabilized by the incubation with culture medium without antibiotics and serum for at least 2 h prior to the transfection. Pre-formed complexes were added directly to the cells and cells were incubated for an additional 6 h. Then, culture medium was replaced with antibiotic and 10% FBS-containing DMEM and incubated for 24 ~ 72 h prior to each experiment.

TB4 was overexpressed by the transfection of cells with pCMV-TB4 or pEGFP-C2-TB4 plasmid DNA, which was accompanied with pCMV-2B or pEGFP-C2 for control group, respectively.

### Gaussia luciferase assay for promoter activity

Pre-designed promoters for TB4 (NM_021109) were obtained from GeneCopoeia Inc. (Rockville, MD, USA). TB4-promoter (HPRM20842) was 1,242 bp (-2,223 ~ -982) upstream from starting codon for TB4 transcription in Homo sapiens X BAC RP11-102M2 (AC139705.4) 36.

HeLa cells were transfected with the TB4-Gluc plasmids using lipofectamine 2000 (Invitrogen, Carlsbad, CA, USA) as described above. Then, cells were incubated for an appropriate time. Secreted Gluc reporter protein was obtained by the collection of culture-conditioned media after the indicated time intervals. Gluc activity of reporter protein was measured by BioLux^®^ Gluc assay kit (New England BioLabs, Ipswich, MA, USA) including coelenterazine as a substrate for Gluc according to the manufacturer's protocol. Luminescence was detected by using Lumet 3, LB9508 tube luminometer (Berthold Technologies GmbH & Co. KG, Bad Wildbad, Germany).

### Western blotting

Cells were lysed in ice-cold RIPA buffer (Triton X-100,) containing protease inhibitor (2 μg/ml aprotinin, 1 μM pepstatin, 1 μg/ml leupeptin, 1 mM phenylmetylsufonyl fluoride (PMSF), 5 mM sodium fluoride (NaF) and 1mM sodium orthovanadate (Na_3_VO_4_)). The protein concentration of the sample was measured using SMART^TM^ BCA protein assay kit (Pierce 23228) from iNtRON Biotech. Inc. (Seoul, Rep. of Korea). Same amount of heat-denatured protein in sodium dodecyl sulfate (SDS) sample buffer was separated in sodium dodecyl sulfate polyacrylamide gel electrophoresis (SDS-PAGE), and then transferred to nitrocellulose membrane by using electro blotter. Equal amount of loaded sample on membrane was verified by ponceau S staining. The membrane was incubated with blocking solution (5% non-fat skim milk in Tris-buffered saline with Tween 20 (TBST)), and then followed by incubation with the specific primary antibodies. Horse radish peroxidase (HRP)-conjugated secondary antibody was used for target-specific primary antibody. Target bands were visualized by the reaction with enhanced chemi-luminescence (ECL) (Dong in LS, ECL-PS250). Immuno-reactive target bands were detected by X-ray film (Agfa healthCare, CP-BU new) or chemiluminescence imaging system Fusion Solo (VilberLourmat Deutschland GmbH, Germany) 36.

### Statistical analysis

Experimental differences were verified for statistical significance using ANOVA and student's t-test. P value of p*<0.05, p** < 0.01 was considered to be significant.

## Results

### DEHP enhanced tumor growth, TB4 expression and primary cilium (PC) formation

**Figure 1:**
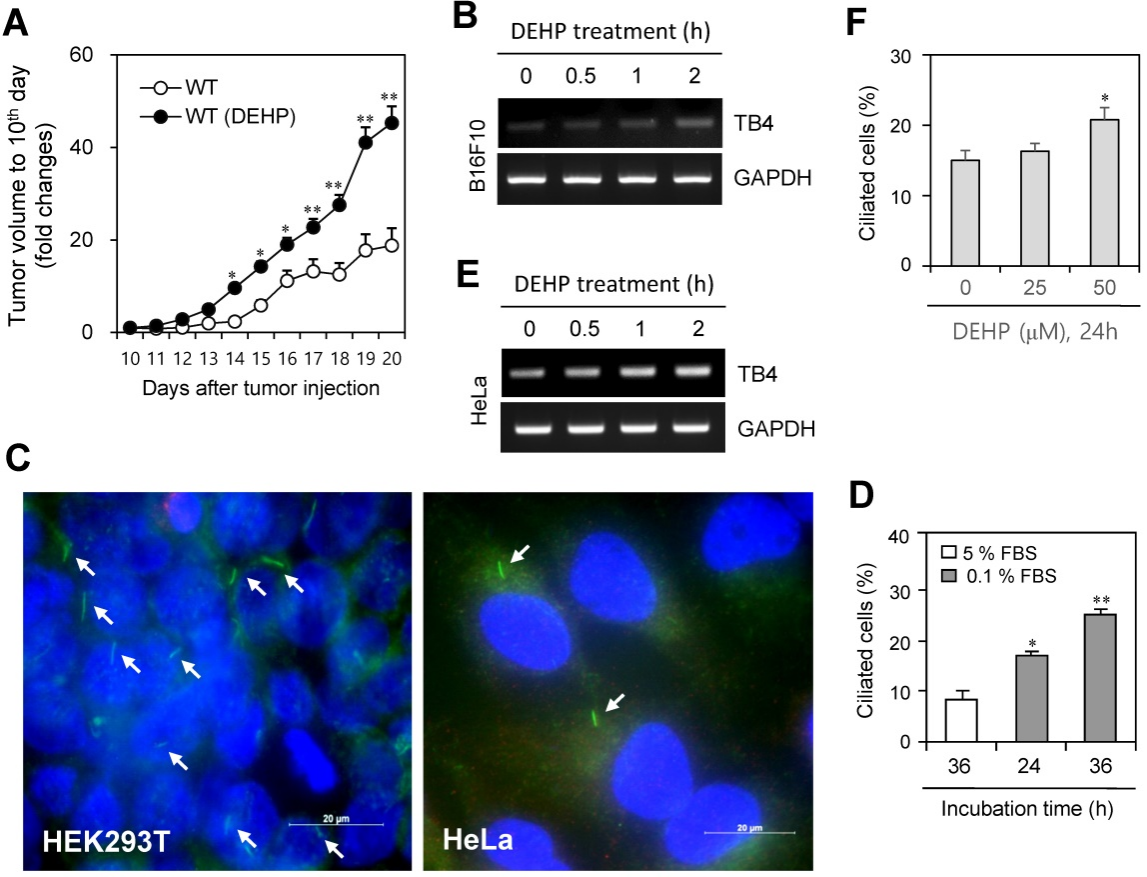
DEHP increased tumor growth, cilia formation and TB4 expression. (A) Six weeks old male mice were pre-exposed to 4 mg/kg DEHP for 21 days and B16F10 mouse melanoma cells were subcutaneously injected into control and DEHP-pre-treated mice. Then, mice were treated with 4 mg/kg DEHP for additional 7 days. Tumor volume changes were daily measured in DEHP-treated group (●) as compared to control (○) group. (B) B16F10 cells were treated with 100 µM DEHP for 0.5, 1, and 2 h. TB4 expression was measured by RT-PCR. (C) HEK293T (left) and HeLa (right) cells were incubated in serum-starved media with 0.1% FBS for 36 h. The cells were fixed and stained with antibody against Ac-tubulin (green). The representative image of primary cilia was observed with 400X magnification under fluorescence microscope. (D) The ciliated HeLa cells in the presence (white) or absence (grey) of FBS were counted (n > 500 cells). (E) HeLa cells were treated with 100 µM DEHP for 0.5, 1, and 2 h. TB4 expression was measured by RT-PCR. (F) HeLa cells were treated with 25 and 50 µM DEHP for 24 h. The cells were fixed and stained with antibody against Ac-tubulin. The ciliated HeLa cells were counted (n > 500 cells). Data in line (A) or bar (D and F) graphs represents the means ± SEM. *p<0.05, **p<0.01; significantly different from control group (A, D, F).

Since di-(2-ethylhexyl) phthalate (DEHP) regulates tumorigenesis 40-44 and TB4 plays a role in tumorigenesis 22-25, we examined the effect of DEHP on tumor growth, primary cilium (PC) formation and TB4 expression. When mice were pre-exposed with DEHP and B16F10 mouse melanoma cells were xenografted into mice, tumor growth was increased in DEHP-pre-treated mice compared to that in DEHP-untreated control mice (Fig. 1a). Tumor volume was about twice on 20^th^ day after tumor injection. When B16F10 cells were treated with 100 μM DEHP for 0.5, 1, and 2 h, TB4 expression was enhanced time-dependently (Fig. 1b). Relatively high frequency of primary cilia was observed by using serum-starved culture condition 58. Then, we also examined PC formation in HEK293T and HeLa cells. Primary cilia were visualized by immunofluorescence staining to acetylated (Ac-) tubulin, a fundamental component of PC structure. PC formation was observed in HEK293T (Fig. 1c, left) and HeLa (Fig. 1c, right) cells under serum-starved media with 0.1% FBS for 36 h. Increase in PC formation was confirmed by counting over 500 cells (Fig. 1d). Based on that TB4 plays a role in PC formation 36, we measured the effect of DEHP on TB4 expression and PC formation in HeLa cells. When HeLa cells were treated with DEHP, we found the increase in TB4 expression (Fig. 1e) and the frequency of primary cilia (Fig. 1f). It suggests that an increase in tumor growth by DEHP pre-exposure might be associated with DEHP-induced TB4 expression and PC formation.

### DEHP-mediated PC formation was regulated by ROS-induced TB4 expression

**Figure 2:**
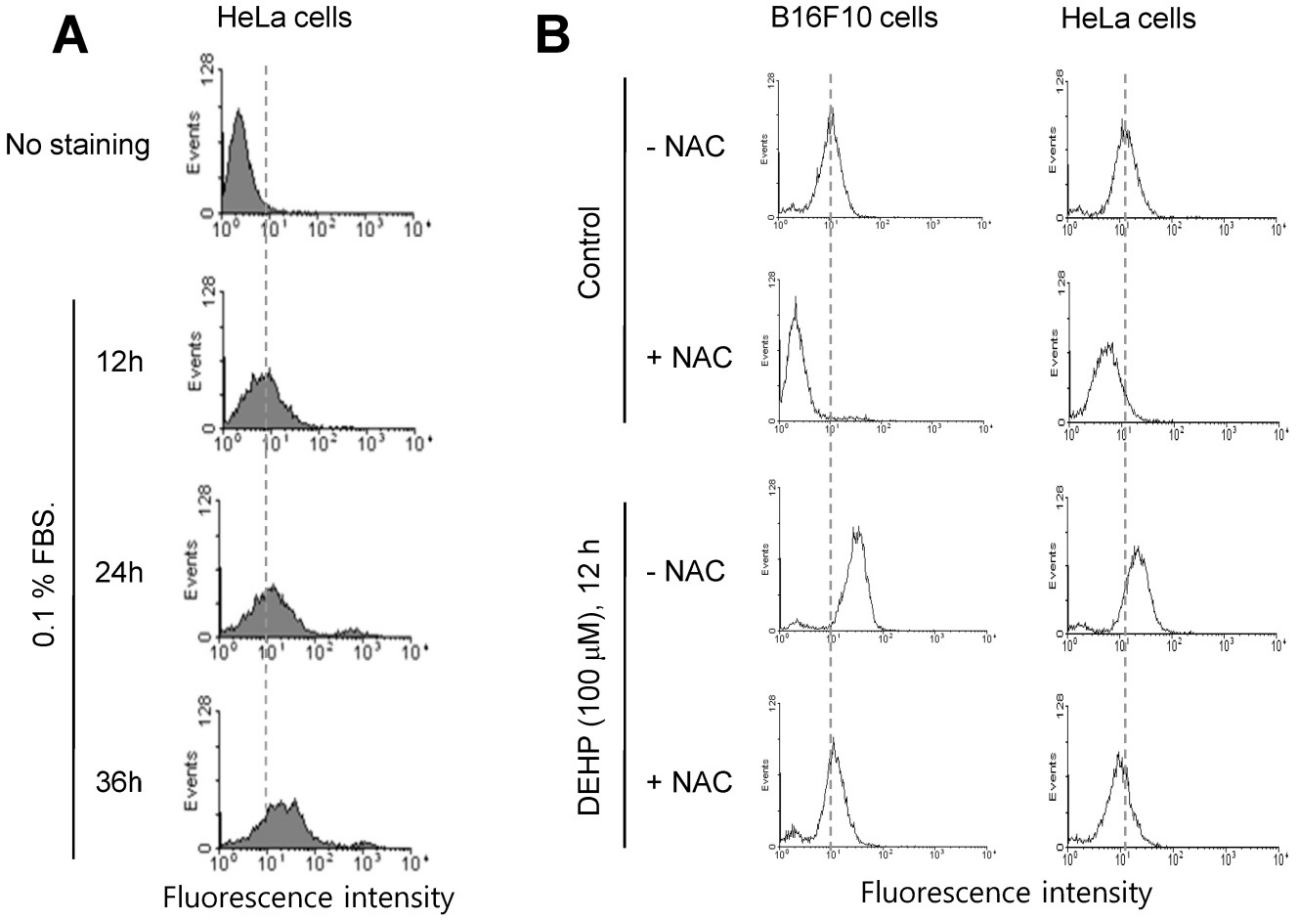
ROS production by DEHP treatment or serum starvation. (A) HeLa cells were incubated with 0.1% FBS for 12, 24 and 36 h. (B) B16F10 and HeLa cells were in cubated with DEHP for 12 h in the presence or absence of N-acetylcysteine (NAC). Cells were treated with DCF-DA and ROS production was measured by FACS analysis (A, B).

Since it has been reported that TB4 is a novel regulator for PC formation 36, we investigated whether TB4 expression could be regulated by any factors influenced by DEHP. Based on that relatively high frequency of primary cilia was observed by using serum-starved culture condition 58, we examined ROS production under serum starvation in HeLa cells. ROS production was increased by the incubation with 0.1% FBS for 12, 24 and 36 h (Fig. 2a). When cells were treated with DEHP, ROS were increased in B16F10 (Fig. 2b, left) and HeLa (Fig. 2b, right) cells. ROS production by DEHP was reduced by the incubation with N-acetylcysteine (NAC) (Fig. 2b). These data suggest that DEHP-induced PC formation could be associated with DEHP-induced ROS-TB4 expression axis.

**Figure 3:**
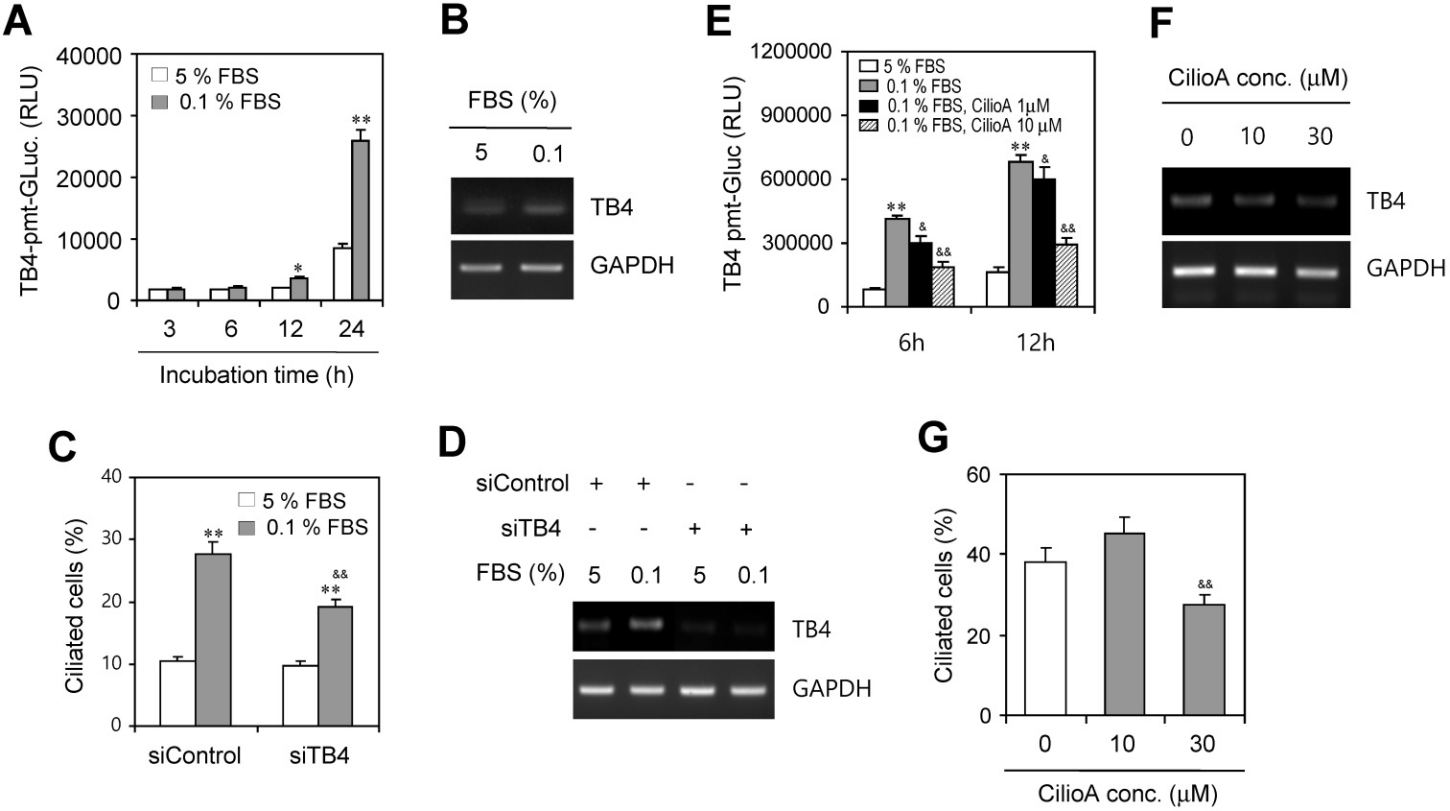
Effect of TB4 on PC formation under serum starvation in HeLa cells. (A-D) HeLa cells were incubated with 5% or 0.1% FBS for up to 24 h after the transfection with pEZX-PG02-TB4-promoter Gaussia luciferase (Gluc) plasmid (A) or after the transfection with AccuTarget™ negative control (siControl) or TB4-siRNA(siTB4) for 24 h (C, D). TB4-Gluc activity in cultured media was measured with luminometer using Gluc substrate (A). The cells were fixed and stained with antibody against Ac-tubulin and the number of ciliated HeLa cells in the presence (white) or absence (grey) of FBS were counted (n > 500 cells) (C). Expression level of TB4 was measured by RT-PCR (B, D). (E-G) HeLa cells were incubated with 0.1% FBS in absence or presence of ciliobrevin A (CilioA) after the transfection with pEZX-PG02-TB4-promoter Gaussia luciferase (Gluc) plasmid. The activity of Gluc in cultured media was measured with luminometer using Gluc substrate (E). Expression level of TB4 was measured by RT-PCR (F). The cells were fixed and stained with antibody against Ac-tubulin and the number of ciliated HeLa cells in the presence (white) or absence (grey) of CilioA were counted (n > 500 cells) (G). Data in bar graphs represents the means ± SEM. *p<0.05, **p<0.01; significantly different from control group incubated with 5% FBS (A, C, E). ^&^p<0.05, ^& &^ p<0.01; significantly different from siControl-treated (C) or CilioA-untreated (E, G) control group with 0.1% FBS.

Then, we examined ROS-mediated TB4 expression by the incubation with 0.1% FBS. HeLa cells were transfected with pEZX-PG02-TB4-promoter Gaussia luciferase (Gluc) plasmid and incubated with 5% or 0.1% FBS for up to 24 h. The activity of TB4-promoter was higher in the incubation with 0.1% FBS compared to the response with 5% FBS (Fig. 3a). TB4 expression was also higher in the incubation with 0.1% FBS compared to the response with 5% FBS (Fig. 3b). We also examined whether PC formation could be regulated by TB4 expression under serum-starved condition. When TB4 expression was inhibited by small interference RNA (siRNA) of TB4, the frequency of primary cilia was increased by serum starvation but it was inhibited by TB4-siRNA (Fig. 3c). Consistently, we confirmed that TB4 expression was increased by serum starvation but it was inhibited by TB4-siRNA (Fig. 3d). Primary cilia-associated expression of TB4 was confirmed by the treatment with ciliobrevin A (CilioA), the inhibitor of ciliogenesis. When cells were transfected with pEZX-PG02-TB4-promoter-Gluc plasmid and incubated with 0.1% FBS in absence or presence of CilioA, the activity of TB4-promoter-Gluc was decreased by the treatment with CilioA (Fig. 3e). Expression level of TB4 was also reduced by the treatment with CilioA (Fig. 3f). Our data also showed that the percentage of primary cilia (~ 38 %) under serum-starved condition was inhibited to ~ 27% by the treatment with 30 μM CilioA (Fig. 3g). It suggests that PC formation could be mediated by the axis of ROS-TB4 expression.

### PC formation was regulated by hydrogen peroxide (H_2_O_2_)-TB4 expression axis

**Figure 4:**
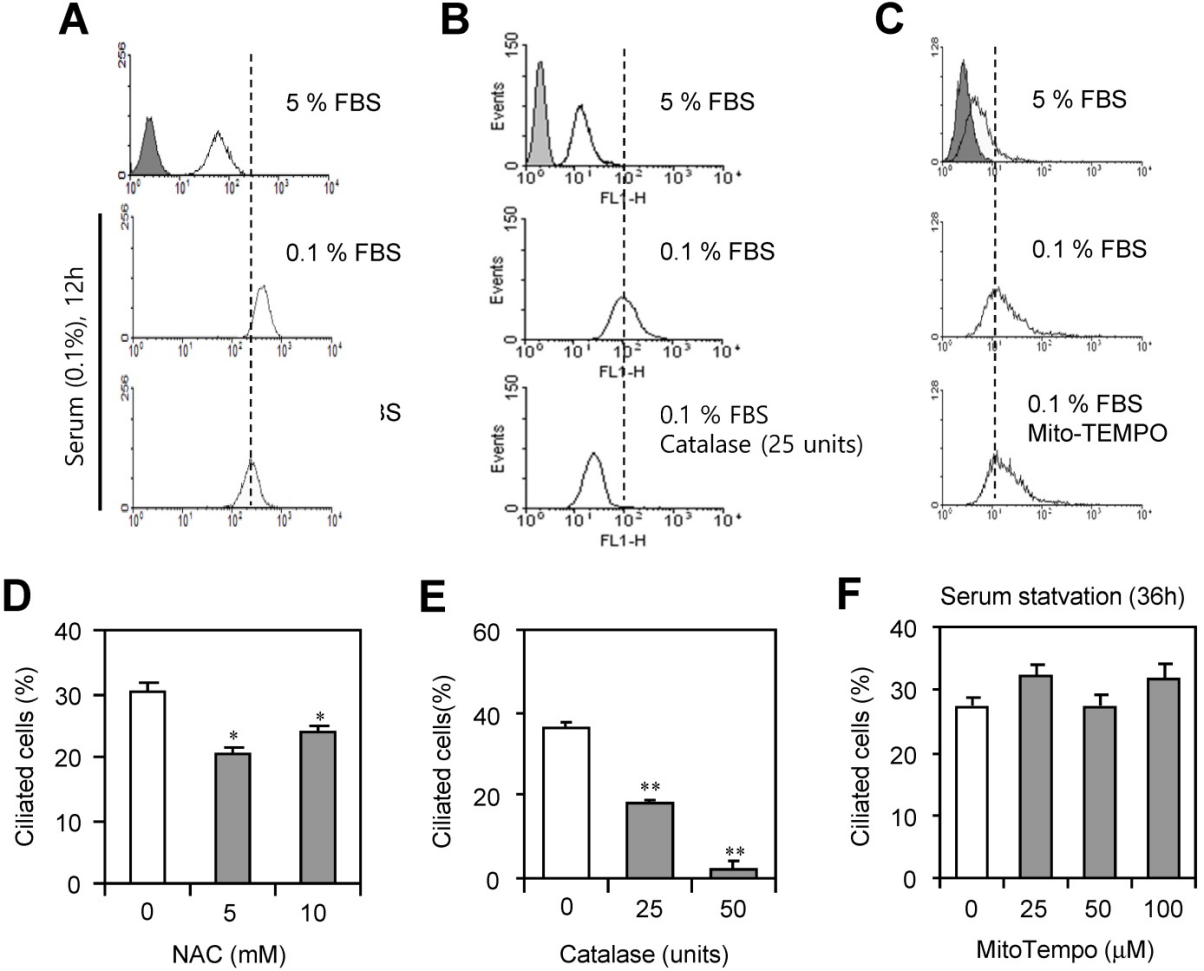
PC formation was reduced by the inhibition of ROS. (A-F) HeLa cells were incubated with 0.1% FBS in the presence or absence of N-acetylcysteine (NAC) (A, D), catalase (B, E) and MitoTempo (C, F). Cells were treated with DCF-DA and ROS production was measured by FACS analysis (A, B, C). The cells were fixed and stained with antibody against Ac-tubulin. The number of ciliated HeLa cells in the presence (white) or absence (grey) of NAC (D), catalase (E) or MitoTempo (F) were counted (n > 500 cells). Data in bar graphs represents the means ± SEM. *p<0.05, **p<0.01; significantly different from control group (D, E).

To examine which factors in ROS are associated with PC formation, cells were incubated with 0.1% FBS in the presence or absence of N-acetylcysteine (NAC), catalase or MitoTempo. ROS production was inhibited by the incubation with NAC, ROS scavenger (Fig. 4a) or catalase to decompose H_2_O_2_ (Fig. 4b). However, no changes in ROS production were detected by the incubation with MitoTempo (Fig. 4c). PC formation was also inhibited by the treatment with NAC (Fig. 4d) or catalase (Fig. 4e). However, no changes in PC formation were detected by the incubation with MitoTempo (Fig. 4f). Data suggest that H_2_O_2_ among ROS could be associated with PC formation in HeLa cells.

**Figure 5:**
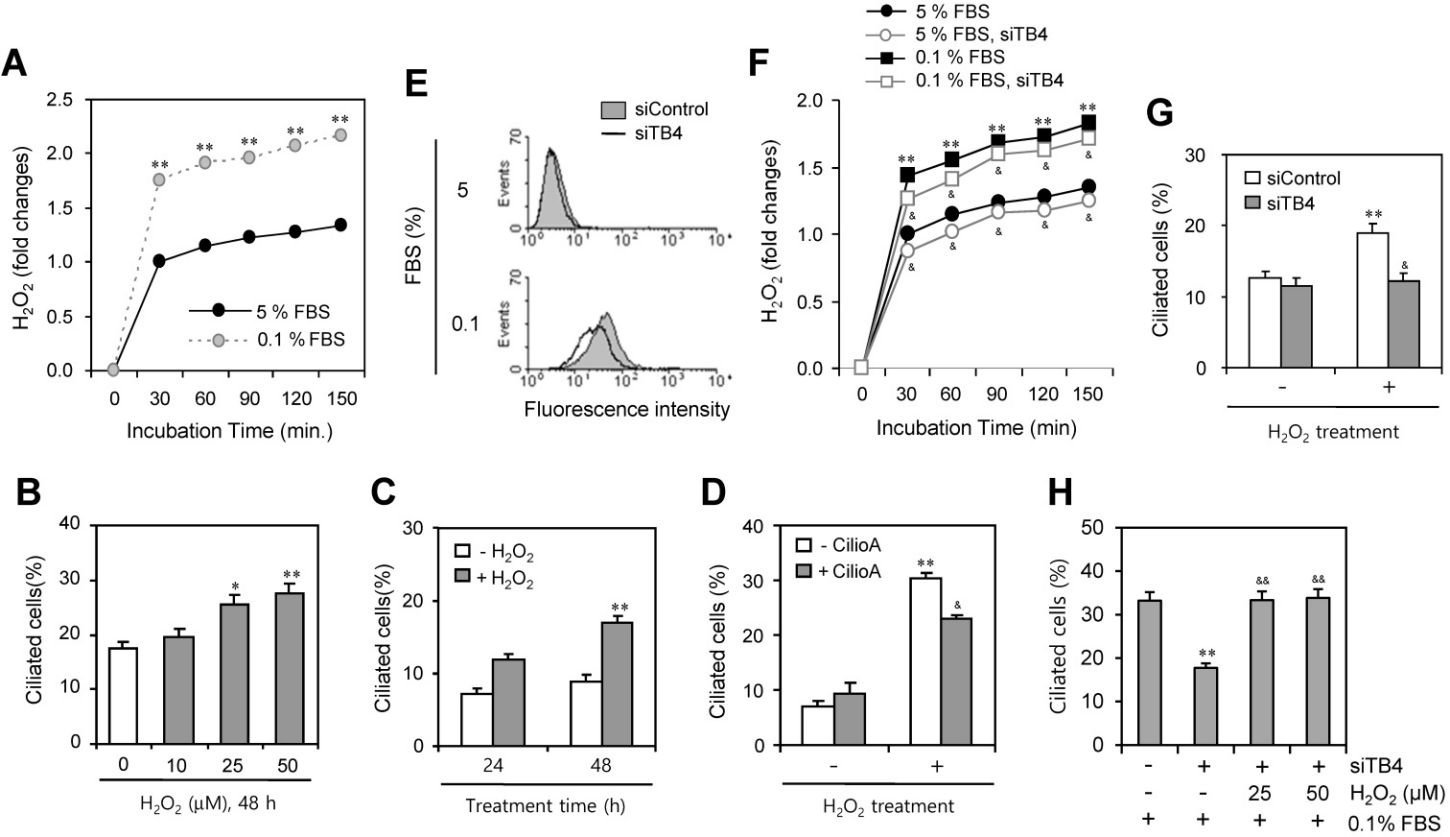
Effect of TB4 expression on H_2_O_2_-mediated PC formation. (A) Cells were incubated with 5 or 0.1% FBS. Then, H_2_O_2_ production was measured with the changes in fluorescence of scopoletin. Then, H_2_O_2_ production was measured with the changes in fluorescence of scopoletin. (B) HeLa cells were treated with various concentration of H_2_O_2_ for 48 h. (C) HeLa cells were treated with 50 μM of H_2_O_2_ for 24 or 48 h. (D) HeLa cells were pre-treated with ciliobrevin A (CilioA) for 2 h and then treated with 50 μM of H_2_O_2_ for 24 h. The cells were fixed and stained with antibody against Ac-tubulin. The number of ciliated HeLa cells were counted (n > 500 cells) (B-D). (E-H) Cells were transfected with AccuTarget™ negative control (siControl) or TB4-siRNA (siTB4) for 24 h. Cells were treated with DCF-DA and ROS production was measured by FACS analysis (E). Cells were incubated with 5 or 0.1% FBS. Then, H_2_O_2_ production was measured with the changes in fluorescence of scopoletin (F). Cells were treated with H_2_O_2_ for 48 h (G). Cells were incubated with 0.1% FBS in the presence of 25 or 50 µM H_2_O_2_ (H). The cells were fixed and stained with antibody against Ac-tubulin. The number of ciliated HeLa cells were counted (n > 500 cells) (G, H). Data in bar graphs represents the means ± SEM. *p<0.05, **p<0.01; significantly different from H_2_O_2_ -untreated (B-D, G) or siControl-treated (H) control group. ^&^p<0.05, ^&&^p<0.05; significantly different from H_2_O_2_-treated and CilioA-untreated (D) or siTB4-treated and H_2_O_2_-untreated (H) control group.

Then, we measured the production of H_2_O_2_ under serum-starved condition. As shown in Fig. 5a, H_2_O_2_ production was significantly increased about twice by the incubation with 0.1% FBS compared to the response with 5% FBS. To confirm the role of H_2_O_2_ on PC formation, cells were treated with various concentration of H_2_O_2_ for 48 h. The number of ciliated HeLa cells were increased dose-dependently from 10 to 50 μM (Fig. 5b) or time-dependent manner from 24 to 48 h (Fig. 5c). When cells were pre-treated with CilioA for 2 h and treated with 50 μM of H_2_O_2_ for 24 h, the number of ciliated HeLa cells were decreased from ~31% to ~23% (Fig. 5d). In addition, we tested the role of TB4 expression on H_2_O_2_ production and the effect of H_2_O_2_-TB4 expression axis on PC formation. ROS production was inhibited by TB4-siRNA significantly (Fig. 5e). When cells were transfected with TB4-siRNA and incubated with 0.1% FBS, H_2_O_2_ production was also reduced by TB4-siRNA significantly (Fig. 5f). Then, when cells were transfected with TB4-siRNA and treated with H_2_O_2_, the number of ciliated cells was also decreased from ~19% to ~12% (Fig. 5g). When cells were transfected with TB4-siRNA under the incubation with 0.1% FBS and then re-treated cells with H_2_O_2_, the number of ciliated cells was increased from 18% to 32% (Fig. 5h). It suggests that PC formation was regulated by the axis of H_2_O_2_-TB4 expression.

### TB4 expression-associated PC formation regulated DEHP-mediated tumor growth

**Figure 6:**
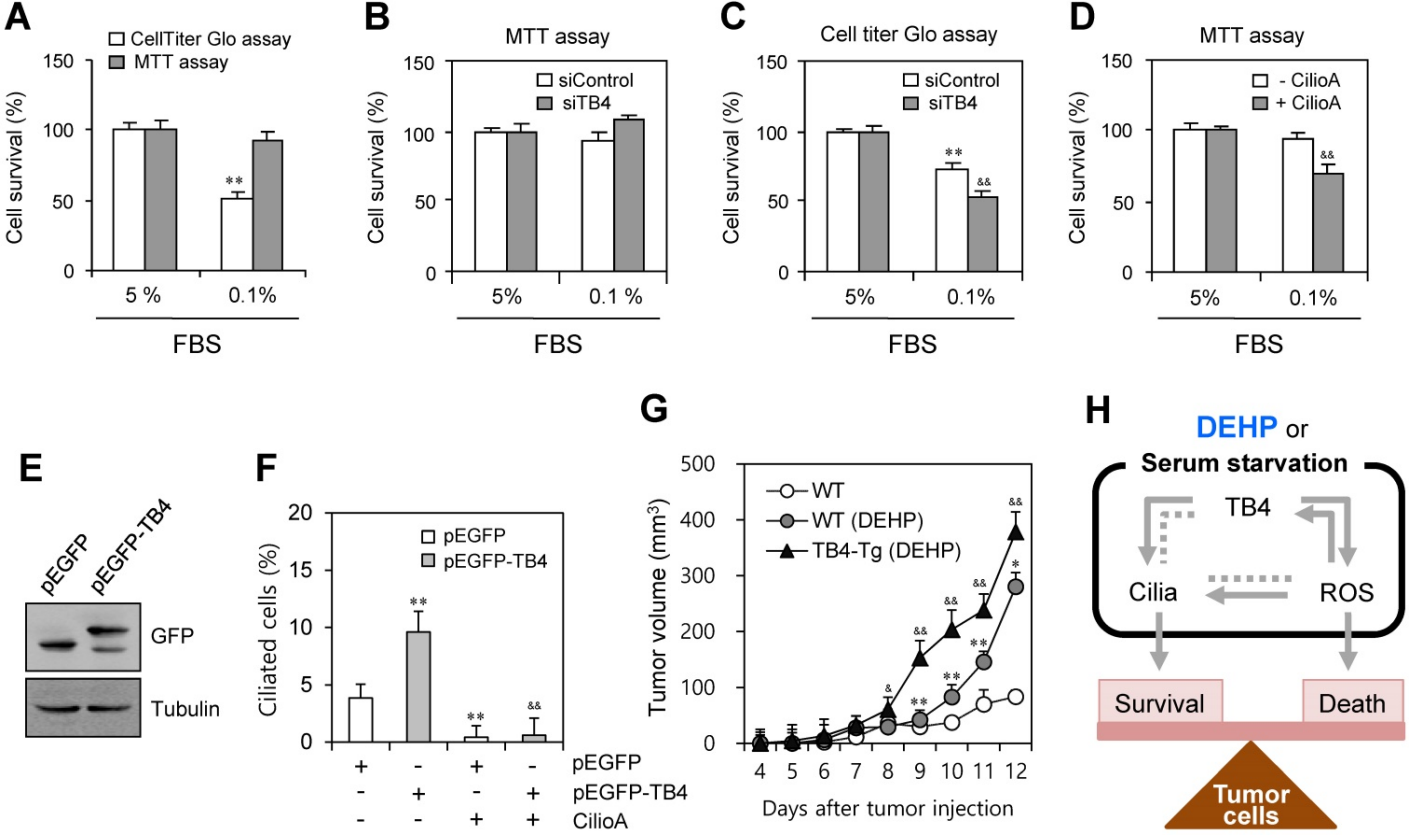
Effect of TB4 on tumor cell death and DEHP-mediated tumor growth. (A-D) Cells were incubated with 5% or 0.1% FBS. Cells were transfected with AccuTarget™ negative control (siControl) or TB4-siRNA (siTB4) for 24 h (B, C). The cells were incubated in the absence or presence of ciliobrevin A (Cilio.A) (D). Percentage of cell survival was measured by MTT assay (A, B, D) or CellTiter Glo assay (A, C). (E, F) HeLa cells were transfected with pEGFP and pEGFP-TB4 plasmid DNA. GFP proteins were detected by western blotting (E). The cells were incubated in the absence or presence of Cilio.A. Then, cells were fixed and stained with antibody against Ac-tubulin. The number of ciliated cells were counted (n > 500 cells) (F). (G) Six weeks old male mice were pre-exposed to 4 mg/kg DEHP for 21 days and B16F10 mouse melanoma cells were subcutaneously injected into control and DEHP-pre-treated mice. Then, mice were treated with 4 mg/kg DEHP for additional 7 days. Tumor volume changes were daily measured in DEHP-treated wildtype (●) and TB4-Tg (▲) groups as compared to control (○) group. Data in bar or line graph represent the means ± SEM. **p<0.01; significantly different from control group with 5% FBS (A, C) or pEGFP-transfected and CilioA-untreated (F) or wildtype (G) control group. ^&^p<0.05; ^&&^p<0.01; significantly different from siControl-treated (C) or CilioA-untreated (D) or pEGFP-TB4-transfected and CilioA-untreated (F) control group or DEHP-treated wildtype mice (G). (H) Scheme for the role of PC formation by ROS-TB4 axis to regulate tumor cell survival and death. DEHP treatment and serum starvation induced PC formation, which is regulated by the axis of ROS-TB4 gene expression in HeLa human cervical cancer cells. Our findings were indicated by grey bold arrows. Unsolved pathway was indicated by grey dotted line.

We examined the effect of TB4 expression and PC formation on tumor cell survival using HeLa cells. When cells were incubated under serum-starved condition with 0.1% FBS, the percentage of cell survival was reduced to ~51% as judged by CellTiter Glo assay. In contrast, no significant changes were detected by MTT assay (Fig. 6a). To confirm the effect of TB4 expression on cell survival, cells were transfected with TB4-siRNA. While no significant changes were detected by MTT assay (Fig. 6b), the percentage of cell survival was reduced from ~68% to ~49% as judged by CellTiter Glo assay (Fig. 6c). The effect of PC formation on cell survival was confirmed by the pre-treatment with ciliobrevin A. The percentage of cell survival was reduced from ~93% to ~65% as judged by MTT assay (Fig. 6d). It suggests that tumor cell survival could be regulated by TB4 expression through PC formation, which is correlated with the changes of intracellular ATP amount rather than a simple mitochodrial enzyme activity.

To test the effect of TB4 expression on DEHP-associated tumor growth via on PC formation, we overexpressed TB4 in HeLa cells or used TB4-transgnic mice. PC formation was re-affirmed by TB4 overexpression using pEGFP-TB4 plasmids (Fig. 6e). The percentage of ciliated cells was enhanced by TB4 overexpression from ~3.7% to ~9.5%, which was reduced to ~0.6% by the treatment with CilioA (Fig. 6f). It implicates once again that tumor cell survival could be regulated by TB4 expression and PC formation. Then, we examined the effect of TB4 expression on DEHP-mediated increase of tumor growth using TB4-transgenic mice. When wildtype and TB4-transgenic mice were pre-exposed with DEHP and xenografted subcutaneously with B16F10 mouse melanoma cells, tumor growth in TB4-transgenic mice was higher than that in wildtype mice compared to that in DEHP-untreated control mice. Tumor volume was respectively about 1.5 or 1.3 times on 11^th^ or 12^th^ day after tumor injection (Fig. 6g). Therefore, these data demonstrate that DEHP-mediated increase of tumor growth might be up-regulated by TB4 overexpression. It also suggests that TB4 could be a novel bio-marker to expect the risk on tumor growth in response to DEHP exposure.

## Discussion

Since primary cilium (PC) transduced various intracellular signaling involved in development and homeostasis, PC abnormality could be major causes of a variety of disorders and diseases including cancers 2-4. PC biogenesis and ROS production are induced by the incubation with serum-starved medium (0.5% FBS) 5-7. Although ROS can be produced by exogenous sources including xenobiotics 12, 13 such as di-(2-ethylhexyl) phthalate (DEHP) 50, no information has been reported about PC biogenesis by xenobiotics. DEHP, one of endocrine disruptors, acts as xenoestrogens which promotes cancer cell metastasis 45 and tumorigenesis 46-48. In addition, little has been known about molecular linkers between PC biogenesis and ROS under xenobiotics-treated conditions. TB4 plays various roles in tumor cell growth 22-25 and metastasis 30-33 in many types of cancer cells. It has been also reported that TB4 play a role in regulating PC formation 36. Therefore, we expected that TB4 could be a possible molecule to link PC formation and ROS production under xenobiotics-treated conditions.

Here, we investigated whether TB4 could be a possible molecule to link PC formation and ROS production and whether DEHP might affect tumor growth by the regulation of PC formation via TB4-ROS axis. Our data demonstrate that PC formation was controlled by Tβ4 through ROS production. Our data also showed that DEHP could augment tumor growth by TB4 overexpression via the increase in PC formation via ROS-TB4 axis.

Although many researches to correlate primary cilia with tumorigenesis have been reported, the effect of ciliogenesis in many types of pre-malignant and invasive tumor cells remains contradictable and elusive 59-61. While PC formation inhibits some tumor cell proliferation 62, relatively high frequency of primary cilia is correlated with some highly proliferative cancer cells such as HeLa cervical carcinoma and MG63 osteosarcoma 58. Therefore, the more study of ciliogenesis in tumor cells would reveal pathological relationship between primary cilia and tumorigenesis or malignancy of tumors. Our data showed that DEHP-mediated increase of tumor growth might be associated with PC formation via TB4 expression (Fig. 1). It is so meaningful to expect the risk of DEHP on tumor growth by PC formation.

Due to that the cilium is assembled in G0/G1 phase and disassembled in S phase and it is disappeared in G2/M phase, primary cilia are obviously observed in cells at G1 phase of cell cycle 63. Since serum starvation induces cell cycle arrest at G1 phase 64, serum starvation is appropriate method to induce PC formation *in vitro*. ROS production could be increased by the incomplete mitochondrial oxidative phosphorylation under serum-starved condition 9. Our data showed that ROS production was also increased by DEHP treatment like by serum-starved condition (Fig. 2). In addition, ROS regulate TB4 expression, leading to PC formation vice versa (Fig. 3). So, it implicates that DEHP could regulate tumor growth by PC formation via ROS production-TB4 expression.

To better understand the detail ROS factor on ciliary formation, we examined the effect of each ROS constituent on PC formation. H_2_O_2_ under serum-starved condition play a main role in PC formation via TB4 expression vice versa (Fig. 4 and 5). Data demonstrate that Tβ4 could be a possible molecule to link ROS production and PC formation. In addition, tumor cell survival was regulated by TB4 expression and PC formation as judged by the measurement of intracellular ATP amount (Fig. 6a-d). Then, more tumor cell death by TB4-siRNA could be reflected to more tumor growth in TB4-transgenic mice pre-exposed to DEHP (Fig. 6g). So, it means that TB4 could be a novel bio-marker to expect the risk of DEHP on tumor growth.

Even though no above explanation is straightforward about the mechanism on tumor growth in DEHP-exposed TB4-transgenic mice, there are several possible signalling molecules to explain how DEHP up-regulate tumor growth by PC formation via ROS (H_2_O_2_) production. Primary cilia are required for tumor growth by smoothened-driven signaling 39, 65, 66. In addition, over 600 proteins are associated in the primary cilia 67. Among them, many molecules are localized on the membrane of primary cilia; ion channels and protein transporters such as transient receptor potential (TRP) ion channels, ion transporters, receptor tyrosine kinase (RTKs), G-protein-coupled receptors (GPCRs) 68, and extracellular matrix (ECM) receptor proteins 4. Primary ciia can transduce diverse cellular signaling mediated by hedgehog (Hh), wigless (Wnt), platelet-derived growth factor (PDGF), hippo (Salvador-Warts-Hippo), JAK/STAT, TRPV4, cAMP/cGMP and mTOR 2, 3. So, it is possible for ROS (H_2_O_2_) to stimulate those signalling pathway for TB4 expression and PC formation. However, it still remains unclear how those proteins regulate tumor growth in DEHP-treated group.

In conclusion, although many questions remain about the mechanisms underlying Tβ4 action on DEHP-induced increase in tumor growth, our findings could be summarized in Fig 6h. It indicated that PC formation by DEHP treatment or serum starvation could be regulated by the axis of ROS-TB4 gene expression and might be associated with the balance for tumor cell survival and death. Our data demonstrate that PC formation could be regulated by TB4 through the production of ROS, especially H_2_O_2_ but not by O_2_^-^. These results might be helpful to better understand molecular linker and hazardous mechanisms responsible for the risk of endocrine disruptor-associated changes. It suggests that TB4 could be a novel bio-marker to expect the risk of DEHP on tumor growth. It also suggests that the individual with higher level of TB4 should be careful not to be exposed repeatedly to DEHP.
